# Bored to Death: Community-Wide Effect of Predation on a Foundation Species in a Low-Disturbance Arctic Subtidal System

**DOI:** 10.1371/journal.pone.0132973

**Published:** 2015-07-17

**Authors:** Eugeniy Yakovis, Anna Artemieva

**Affiliations:** Invertebrate Zoology Department, St. Petersburg State University, St. Petersburg, Russia; University of Waikato (National Institute of Water and Atmospheric Research), NEW ZEALAND

## Abstract

The strength of top-down control by consumers is predicted to decrease with latitude, but most data confirming this assumption come from latitudes <60°, while empirical studies of predation in sub-arctic and arctic marine habitats are few. A barnacle *Balanus crenatus* is a native foundation species in the shallow subtidal of the White Sea (65° N), hosting a diverse (250+ species) assemblage of macrobenthic organisms. On mixed sediments live barnacles share primary substrates (shells and gravel) with numerous empty barnacle tests, 7% of which had drill holes of an unidentified origin. We manipulated the densities of (i) adult muricid whelks *Boreotrophon clathratus* (of previously unknown feeding habits), to check if they prey on barnacles, (ii) other predators to reveal their effect on juvenile *Boreotrophon*, and (iii) empty tests to assess the community-wide effect of predation on barnacles. The abundance of drilled empty tests in the field correlated with that of *Boreotrophon*. A year-long caging experiment clearly confirmed predation, showing the highest barnacle mortality and proportion of drilled tests in whelk enclosures, and the lowest — in predator exclosure treatments. *Boreotrophon* preferred the barnacles attached to conspecifics to those from primary substrates. Because of its scarcity *Boreotrophon* had a minor direct effect on barnacle abundance in the field. Yet, initially defaunated empty tests and live barnacles developed markedly different macrobenthic assemblages, suggesting a strong indirect effect of the predation. Juvenile *Boreotrophon* were 5-6 times less abundant in open and partial cages than in exclosures and enclosures, which indicates that the recruitment and, consequently, the abundance of *Boreotrophon* and its predation on *Balanus* are top-down controlled by apex predators. In contrast, in tropical and temperate intertidal the predation on barnacles is stronger and primarily limited by environmental stress and prey availability.

## Introduction

Interspecific interactions, particularly predation, and top-down control of community structure are considered to weaken with latitude. Despite the growing empirical evidence [1], several studies do not support this assumption [2–3]. To our knowledge, the data confirming the latitudal predation strength gradient originate from the studies geographically scaled between temperate and tropical zones [1,4–6]. However, experimental studies on predation at higher latitudes, especially in sub-arctic and arctic marine habitats, are few [7–8], and the resulting knowledge gap restrains the generalization of global-scale ecological patterns.

Marine benthic communities are often shaped by habitat-forming foundation species that provide biogenic structure which numerous small secondary space holders depend on. Keystone predators that control these influential habitat modifiers affect entire communities disproportionally to their own abundance [9]. On mixed sediments in the White Sea shallow subtidal around Solovetsky islands (65° N) acorn barnacles *Balanus crenatus* monopolize hard substrates scattered on muddy bottom, which are primarily empty shells of a Greenland cockle *Serripes groenlandicus* [10]. At a depth of 12 m the disturbance level is low enough for the epibenthic patches based on cockle shells sized about 40 cm^2^ to persist for years [11]. As in many other habitats dominated by barnacles [12–13] the cover they make up partly consists of empty tests remaining after barnacles' death. Both live *B*. *crenatus* and their empty tests host a highly diverse assemblage of mobile (152 species) and sessile (134 species) macrobenthic organisms [10,14] which is numerically comparable to cryptofauna of tropical coral reefs in terms of species richness [15]. Barnacle cover greatly increases the surface area and complexity of hard substrates available for other taxa, including subdominant solitary ascidians and red algae. *B*. *crenatus* itself utilizes the surface of conspecifics as well, developing multi-tier clusters. The proportion of empty tests in a barnacle patch correlates with relative abundances of different species found there [10,14], but the causal relationship underlying this pattern is unclear. Sources of barnacle mortality, contributing to the supply of their empty tests are also unknown.

According to the studies in tropical and temperate rocky intertidal, empty tests of adult barnacles, including those left by predators, compose a microhabitat favourable for herbivorous snails [16] and barnacle recruits [13,17]. As a result, empty tests can mediate community-wide indirect effects of predation on barnacles while holding primary substrate space. In the intertidal, empty tests primarily attract specific fauna since they ameliorate environmental stress, particularly providing refuge from wave action and altering humidity at low tide [16]. Both these mechanisms are apparently irrelevant in low-disturbance subtidal habitats. Instead, empty tests here may affect the adjacent macrobenthic assemblages by providing more empty space and possibly higher capability for sediment accumulation than live barnacles, and lacking their self-cleaning, feces production, food depletion and flow modification by suspension feeding.

**Fig 1:**
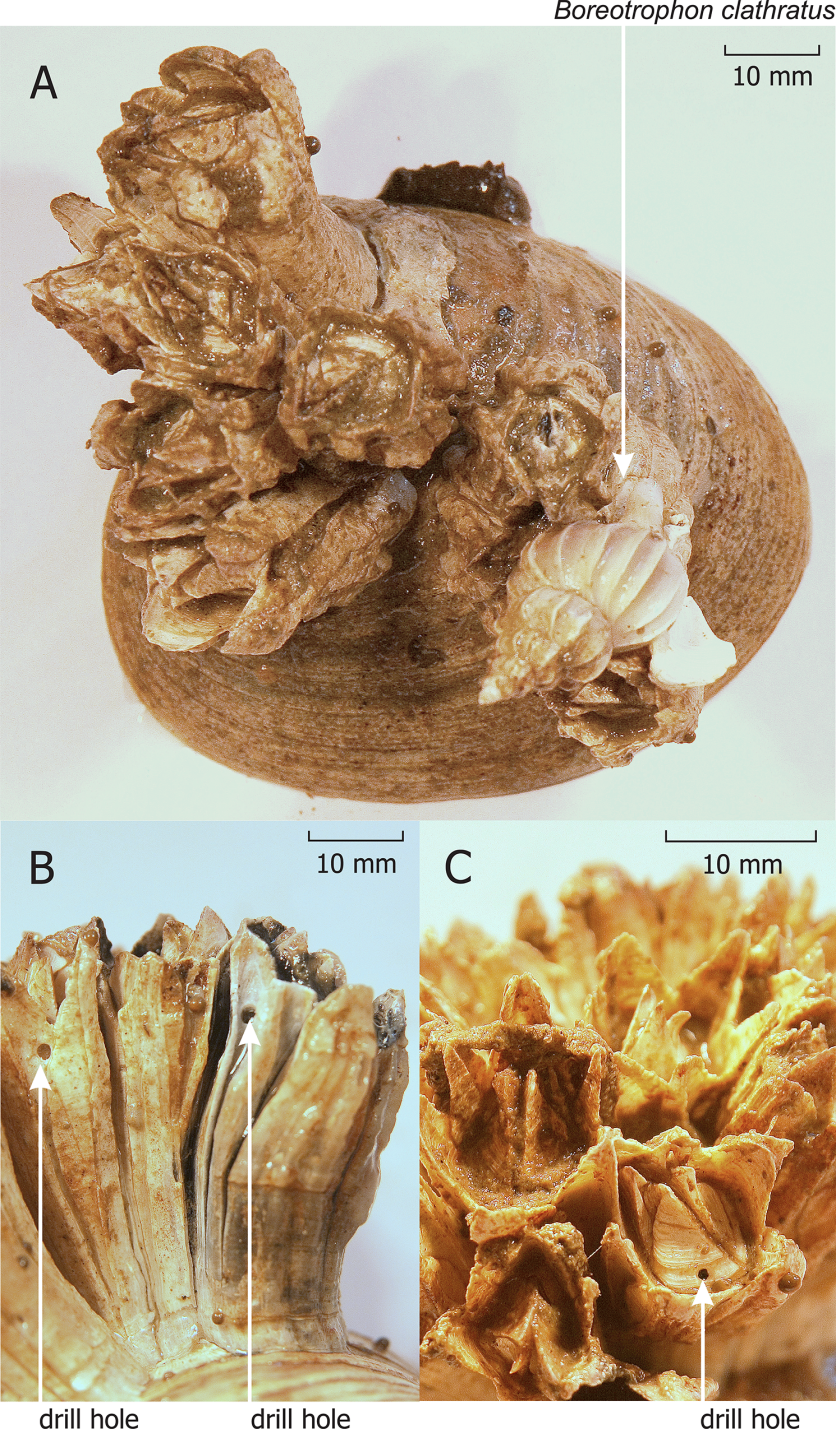
*Boreotrophon clathratus* and drill holes on *Balanus crenatus*. (A) *Boreotrophon clathratus* on *Balanus crenatus* attached to a *Serripes groenlandicus* empty shell. Drill holes on a lateral (B) and opercular (C) shell plates of *Balanus crenatus* empty tests.

Our unquantified field observations show that *B*. *crenatus* empty tests frequently have holes completely through them (Fig 1B and 1C), apparently indicating the predation by a boring muricid or naticid snail. Incomplete drillings (likely the traces of failed attacks) also occur both on live barnacles and their empty tests. In the White Sea, the only known specific predator of barnacles is a nudibranch *Onchidoris bilamellata* [18], neither capable of drilling nor noticeably abundant at our research sites near Solovetsky Islands [19]. None of the species previously recorded in the White Sea are reported to drill barnacle shells there or elsewhere. The most likely predator causing the holes was a clathrate trophon whelk *Boreotrophon clathratus*, the only locally common muricid gastropod (Fig 1A). Muricid gastropods from various genera prey on barnacles by drilling their shell plates (reviewed in [20]), and the cylindrical form of the drill holes we observed also suggested their muricid origin [21]. Yet, the studies on *Boreotrophon* spp. feeding are scarce and only reported them to prey on bivalves [22–24].

In this study we combined field observations and manipulative experiments to examine the relationship between *Boreotrophon clathratus* and *Balanus crenatus*, the indirect community-wide effect of such a relationship, and the factors that may control its strength. Although the high latitude predicts low predation, we suggested that the low abiotic disturbance level (see [25]) in a subtidal habitat and a strong facilitator being the prey might lead to substantial top-down control within the system studied. We predicted that (1) *B*. *clathratus* preys on *B*. *crenatus* by drilling through their shell plates (which leaves empty tests), and that predation strength would be related to the abundance of the former. We also predicted that (2) live *B*. *crenatus* and their empty tests support different macrobenthic assemblages so that the predation on barnacles indirectly affects the whole community, and that (3) the abundance of *B*. *clathratus* is controlled by predation on their juveniles.

## Materials and Methods

### Sampling

To assess the pattern of predation in the field SCUBA divers sampled two 12 m deep subtidal sites near Solovetsky Island (Onega Bay, the White Sea) at 65°01.180’N, 35°39.721’E (1) and 65°01.117’N, 35°40.039’E (2) in July and early August 2010–2013. The first site was also defined as 'Site 1' in our previous communications [10], [14], [26]. The sediment was muddy sand with 34±5 m^–2^ empty mollusc shells (including shell fragments) and 5±1 m^–2^ gravel visible on the bottom surface at Site 1 (7±2 m^–2^ shells and 10±2 m^–2^ gravel at Site 2). These substrates were typically covered by barnacles and their empty tests. Most of the empty shells belonged to a Greenland cockle *Serripes groenlandicus*. Bottom temperature in July was 5–9° C, and salinity, according to earlier observations, was 24.4–27.6 ppt [19].

Divers collected all visible hard substrates (shells and gravel) and live whelks *Boreotrophon clathratus* from inside 1 m^2^ frames placed haphazardly on the bottom. Each frame was sorted as a single sample. We examined these samples in the laboratory and documented aperture length along the rostro-carinal axis (accurate to 1 mm; hereafter 'size'), growth bands count, underlying substrate, presence and location of complete (through) and incomplete drill holes for each individual *Balanus crenatus* or its empty test found. All the live barnacles in a sample were wet weighed (accurate to 1 g), and 300–350 random live barnacles in each sample were weighed individually (accurate to 0.001 g) to develop a relationship between the size and weight (2964 individuals in total, see below). Multiple complete drillings of a single empty test were extremely rare and counted as a single drilling. *B*. *clathratus* were individually measured (shell height accurate to 1 mm; hereafter 'size') and wet weighed accurate to 0.001 g. A *Balanus crenatus* test consists of fused immobile wall plates and 4 separate opercular mobile plates, namely paired *tergum* and *scutum*. Empty tests found in the field often lack opercular plates. Since uneven preservation rate of different plates might bias the estimates of predation based on drill hole counts, in 2011–2013 we recorded the number of opercular plates (0 to 4) preserved within each empty barnacle test.

In total, 9 such 1 m^2^ frames were sampled. To avoid unneccessary processing of numerous recruits since 2012 we recorded small (≤1 mm) empty barnacle tests only within a random 0.25 m^2^ square subsample of the 1 m^2^ frame (see Table 1 for details).

**Table 1:** Field abundance of live and dead barnacles *Balanus crenatus* and whelks *Boreotrophon clathratus*.

			Live *Balanus crenatus*										
Sampling details				Abundance by aperture length per m^2^				*Boreotrophon clathratus*		Empty *Balanus crenatus* tests by aperture length per m^2^: total vs drilled (in parentheses)			
Date	Site	Area (m^2^)	Total weight, g/m^2^	1 mm	2–4 mm	5–9 mm	10+ mm	Weight, g/m^2^	Ind. per m^2^	1 mm	2–4 mm	5–9 mm	10+ mm
14.07.2010	1	1.00	977	22	27	285	358	2.981	6	47 (2)	126 (9)	70 (16)	80 (9)
21.07.2010	1	1.00	529	96	110	414	172	2.777	3	112 (0)	303 (3)	116 (5)	71 (2)
24.07.2011	1	1.00	713	189	168	336	259	1.259	4	12 (1)	57 (4)	76 (13)	107 (12)
28.07.2012	1	0.25+0.75^a^	709	1755	253	248	255	2.844	13	234 (72)	96 (8)	52 (6)	110 (11)
05.08.2012	1	0.75^b^	517	1737	395	157	145	0.895	12	n/a	49 (8)	73 (4)	63 (3)
03.08.2012	2	0.25+0.75^a^	202	358	100	89	89	1.498	2	36 (0)	14 (0)	52 (2)	42 (3)
03.08.2012	2	0.25+0.75^a^	248	1000	302	208	91	0.005	2	124 (16)	57 (0)	33 (2)	14 (0)
29.07.2013	1	0.25+0.75^a^	720	3905	48	124	279	1.094	3	456 (4)	85 (3)	89 (3)	94 (4)
03.08.2013	1	0.25+0.75^a^	354	2182	31	132	125	5.736	4	96 (0)	75 (4)	64 (7)	75 (2)

Only the empty tests with complete through drillings considered 'drilled'.

^a^ Empty barnacle tests smaller than 2 mm and drill holes thereon recorded from a random 0.25 m^2^ subsample of a 1.00 m^2^ sample

^b^ Drill holes on barnacle tests smaller than 2 mm not recorded

No specific permissions were required for the accomplished field sampling activities by St.-Petersburg State University. The field studies were conducted exclusively in public and not protected marine areas. The field studies did not involve any endangered or protected species. The research was performed in full compliance with federal law of the Russian Federation 'On Environmental Protection' (Federal law 7-FZ from January 10, 2002). Animal capturing, handling and killing was designed to avoid distress and unnecessary suffering.

### Field experiment on the effect of *Boreotrophon clathratus* on *Balanus crenatus*

To test the effect of *Boreotrophon clathratus* on barnacles we arranged field caging experiments. SCUBA divers collected empty *Serripes groenlandicus* shells with 10–30 adult (4 or more growth bands, sized at least 5 mm, 10.5±0.2 mm average size) barnacles within 200 m^2^ around Site 1 in July 2009, 2010 and 2011. From these substrates we manually removed all the macrobenthic organsims >0.3 mm under a binocular microscope, mobile and sessile, except the live adult barnacles. For the latter, we selected the threshold of 5 mm size to ensure that in a year we would not confuse the barnacles that initially entered the experiment with the later arriving recruits. Most barnacles resided directly on cockle shells (1^st^ tier), though about 8% grew on conspecifics (2^nd^ tier). We attached shells with barnacles to the bottom of 300×375×70 mm plastic cages, covered with 2.5 mm nylon mesh. Before applying the mesh we photographed each cage to document the initial number and positions of barnacles ≥5 mm. We tested the effect of whelks on barnacles by four treatments: (i) full cages (= predator exclosures), (ii) open cages (no mesh), subject to normal predation, (iii) caged barnacles with whelks added (= predator enclosures) and (iv) partial cages to control for the effect of caging, which were like full cages except that the mesh had two side windows 60×20 mm each. Every cage initially contained on average 45±4 live adult *B*. *crenatus* in 2–3 clusters. Their weight was 78.7±9.5 g per cage (700 g per m^2^). Within a particular year cages were randomly assigned to treatments so that the total number of adult barnacles per cage was similar between the treatments (ANOVA, F_3,20_ = 1.42, p = 0.265). To the whelk enclosures we added 6–8 adult (≥8 mm) *B*. *clathratus* per cage. The size threshold of 8 mm was selected so that the whelks could not pass through the 2.5 mm mesh. We used a higher than observed density of whelks (per barnacle) to increase the likelihood of detecting the predation and assessing any selectivity depending on individual properties of the prey. We exposed the cages anchored to the bottom at Site 1 for one year starting July 2009, 2010 and 2011, two cages for each of the four treatments per year (24 cages in total). Permanent ice cover from December to May and hard storms in October-November limit the accessibility of the shallow subtidal around Solovetsky Islands to June-September. This complicates seasonal manipulations and determined the annual time-scale for the present study. Upon retrieval after exposure, all the live barnacles, their empty tests, and whelks from every cage were examined similarly to those from field samples (see above), except that we weighed *B*. *clathratus* individually in 2011–2012, and in 2010 we only recorded their total weight per cage. We then estimated the individual weight (in g) from size (in mm) according to the relationship Weight = 0.0028 · Size^2^ – 0.0304 · Size + 0.083 (R^2^ = 0.96, n = 57), based on the measurements of the whelks from field samples and cages. Live barnacles in all the treatments were also weighed per cage, and their individual weight (in g), was estimated from size (in mm) according to the relationship Weight = 0.0032 · Size^2.6^ (R^2^ = 0.73, n = 2964), based on individual measurments in the field samples. Only the complete through perforations on the empty tests were counted as drill holes.

In addition, for each live barnacle or an empty test we recorded lateral contiguity percentage as a fraction of their outer wall plate area closely adjacent to or fused with neighbors. We expected this parameter to reflect the vulnerability of an individual to an attack by drilling predators. We also counted and weighed crabs and shrimps found in the cages since they presumably could pass through the 2.5 mm mesh as juveniles, and get trapped inside as they grow.

Throughout the experiments (2009–2012), a relatively heavy barnacle settlement happened only once, in 2012. Here, we opted not to perform a detailed analysis of barnacle recruits (<5 mm) abundance. We also did not manipulate the presence of small *B*. *clathratus* and shrimps, which might prey on barnacle recruits.

In addition to the four abovementioned main treatments, we added a starfish enclosure cage in 2010–2011 with 13 adult *Henricia* sp. individuals (total weight 5.3 grams) and 3 spider crab enclosure cages, 1 in 2010–2011 and 2 in 2011–2012 with 2 adult *Hyas araneus* individuals each (total weight 7.3 grams per cage). Because of the logistic constraints imposed by the limited number of substrates with barnacles we could initially collect, we had no more replicates for these treatments. Consequently, we did not include starfish and crab cages in any statistical analysis. We, however, used these data as supplementary when discussing possible sources of mortality in adult barnacles and juvenile whelks.

### Field experiment on assemblages associated with live barnacles and their empty tests

Cockle shells with adult barnacles were collected and processed in the same way as for the previously described experiment (see above) around Site 1 in July 2010 and 2011. In the laboratory, all the macrobenthic organsims except the live adult barnacles (on average 20±2 individuals per shell) were removed from these substrates. Afterwards, barnacles on a random half of the shells were transformed into empty tests ('ET' treatments) by removing soft tissues and mobile shell plates, while the other half ('LB' treatments) was left alone with live barnacles. We attached ET and LB shells in alternating sequences to 390×280 mm plastic grids (3×ET and 3×LB per grid) and anchored them to the bottom at Site 1 for one year starting July 2010 and 2011 (6 replicate grids in total, 2 in 2010 and 4 in 2011).

Since some empty barnacle tests collapsed by the end of the experiments, ETs became generally smaller than LBs. To even their size, we selected one ET with the smallest count of empty tests left in each grid, and excluded it from the further analyses. Out of the 6 ETs excluded 5 had 9 or fewer empty tests and one had 12. One LB had been also accidently destroyed during the manipulations. The remaining treatments had no significant difference between the average numbers and sizes of barnacles in LB and empty tests in ET (Student T-test, p = 0.062 and 0.430, respectively). The total numbers of the remaining LB and ET treatments used in the analyses were 17 and 12, correspondingly.

After a year of exposure we collected the experimental substrates with live barnacles and empty tests and examined them in the laboratory as separate samples, identifying (generally to species level) and counting every macrobenthic organism sized 0.5 mm and larger. In 2011, we additionally weighed the sediment washed out from most of the experimental substrates (dried on paper towels for 36 hours) to compare sediment particles accumulation rate by live and dead barnacles.

### Data analysis

We used Pearson correlations with Bonferroni correction for multiple comparisons to link the abundances of live *B*. *crenatus*, their empty tests with and without drill holes and *B*. *clathratus* in the field samples. The results of the caging experiments were analyzed with type III sum of squares 2-way ANOVA followed by Tukey HSD post-hoc tests for pairwise means comparison. The factors were Treatment (fixed with 4 levels: open cage, partial cage, exclosure cage, whelk enclosure cage) and Year (random with 3 levels: 2009–2010, 2010–2011, 2011–2012). The following variables were examined: numbers of small (≤7 mm, as these could pass through 2.5 mm mesh, see below) and large (≥8 mm) *B*. *clathratus* individuals per cage, total mortality of barnacles ≥5 mm (the number of empty tests in a cage by the end of experiment divided by the total number of live barnacles and empty tests), the mortality of barnacles ≥5 mm not associated with drilling (same as the previous variable, except that the number of empty tests without complete drill holes was used in the numerator), the mortality of barnacles ≥5 mm associated with drilling (same as the previous one, but the number of empty tests *with* complete drill holes used as the numerator), and the total number of drilled empty tests in a cage. The variables included in the analysis were checked for homogeneity of variances (Cochran's test, p > 0.05), and square root transformed to eliminate heterogeneity where needed. The resulting variances were homogeneous in all the variables. Separate one-way ANOVA with the factor Treatment was performed on the fraction of empty tests with complete drill holes, since some cages had no empty tests at all, and the effect of Year could not be estimated. This analysis was unbalanced, and despite the transformations applied the variances remained heterogeneous. However, as there was no significant correlation between means and variances, we found the results interpretable.

Macrobenthic assemblages from the patches of initially defaunated empty barnacle tests and live barnacles were analyzed in terms of species richness and log-e based Shannon species diversity with type III sum of squares 2-way ANOVA followed by Tukey HSD post-hoc tests for pairwise means comparison, where the factors were Live (fixed, two levels: live barnacles and empty tests) and Grid (random, six levels: grid numbers 1–6). Variances were homogeneous (Cochran's test, p > 0.05). Species composition and abundances in these assemblages were examined using 2-way permutational multivariate analysis of variance (PERMANOVA, [27]) on Bray-Curtis similarities with the same factors Live and Grid as in the previously described ANOVA. Abundances were standardized by the number of adult barnacles or their empty tests on a substrate and fourth root transformed prior to analysis. We used non-metric multi-dimensional scaling (nMDS) on Bray-Curtis similarities to produce a two-dimensional ordination plot visualizing the relationships between the assemblages associated with live barnacles and their empty tests. The taxa that most contributed to average measures of dissimilarity between these assemblages were identified using SIMPER [28]. For each of these taxa we ran a type III sum of squares 2-way ANOVA followed by Tukey HSD post-hoc tests with the factors Live and Grid (see above) on abundances (transformed, where needed, to achieve homogeneity of variances [Cochran's test, p > 0.05]) to test their preference for either of the microhabitats.

For all tests significance level was 0.05 and means were ± S.E. unless stated otherwise.

## Results

### Field sampling

The results of the sampling are summarized in Table 1. On average there were 1750±405 live individuals of *Balanus crenatus* per square meter of the bottom, of which 267±35 had aperture length of 5 mm and larger (hereafter 'adults'). Mean biomass of barnacles was 552±85 g·m^-2^ (in 2012, when we sampled both sites, it was 613±48 g·m^-2^ at Site 1 and 225±12 g·m^-2^ at Site 2). Mean abundance of empty barnacle tests was 384±71 m^-2^ (29±8% of the abundance of live barnacles). Their fraction was insignificantly (37±4%) higher in adults (pairwise Student T-test p = 0.274).

**Fig 2:**
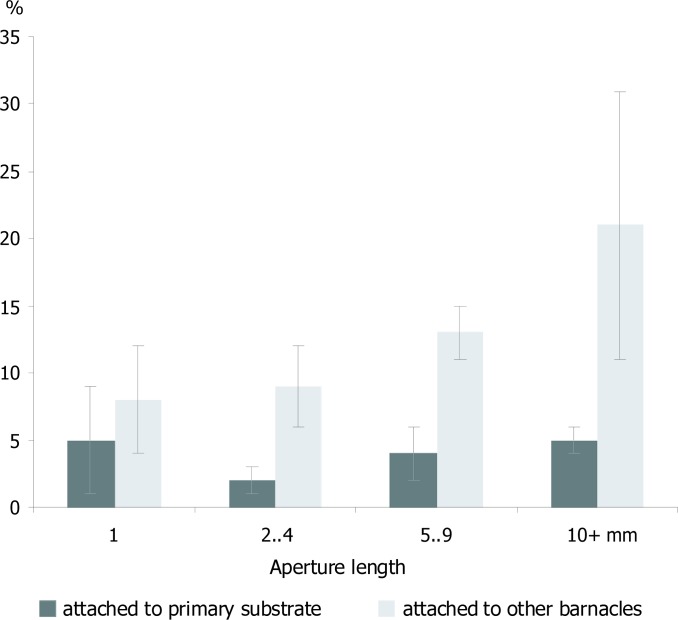
Average proportion of drilled empty tests by size and substrate in the field. Error bars denote S.E.

In total, 7.0±1.4% of the empty tests found in the field had drill holes completely penetrating the shell. This proportion was similar for the large (≥5 mm) and small tests (7.7±1.6% and 6.4±2.1%, respectively; pairwise Student T-test p = 0.312). Most live barnacles resided either on primary substrate, e.g. empty bivalve shells and gravel (55±5% of individuals, hereafter 'first tier') or on conspecific adults, live or dead (40±5%, hereafter 'second tier'), and so did the empty barnacle tests (59±5% and 36±5%, correspondingly). Empty tests from the second tier carried significantly more drill holes than those from the first one (12±2% vs 4±1%, pairwise Student T-test p = 0.001), and the disproportion increased with the size of the tests (Fig 2). Only about 15% of empty tests ≥5 mm preserved any mobile plates, and thus most of the drill holes found were located on the lateral immobile ones (Table 2). Two times we observed a second-tier empty test drilled through a bottom plate and a wall of the underlying first-tier empty test. Some live barnacles ≥5 mm had incomplete blind drill holes (3.5±0.7% of the population) and other 1.1±0.2% were non-fatally drilled through the upper parts of the wall plates around the orifice. Thus, in total, 4.6±0.6% of live barnacles ≥5 mm carried the traces of failed attacks.

**Table 2:** Field preservation of opercular plates (*tergum* and *scutum*) in empty barnacle tests with aperture length not less than 5 mm by location of drill holes (if any).

	Shell plate drilled		
	None	Wall	Opercular
No opercular plates preserved	79.4 ± 3.3 %	5.2 ± 1.4%	-
Some (1–3) opercular plates preserved	2.3 ± 1.1%	0.1 ± 0.1%	0.0 ± 0.0%
All the opercular plates preserved	11.9 ± 3.3%	0.4 ± 0.2%	0.8 ± 0.4%

Sampled obtained in 2011–2013 (n = 7 samples) used. Total number of tests examined was 909.

We found 5.44±1.40 *Boreotrophon clathratus* individuals per square meter with an average biomass of 2.121±0.564 g·m^-2^. The abundance and biomass of *B*. *clathratus* was not significantly correlated neither with those of *Balanus crenatus*, nor with the total number of empty barnacle tests per square meter. The number of drilled barnacle tests, however, had high significant positive correlation with the abundance of *B*. *clathratus* (Table 3).

**Table 3:** Pearson correlations between the parameters of *Balanus crenatus* and *Boreotrophon clathratus* in the field samples.

*Balanus crenatus*	*Boreotrophon clathratus*	
	Abundance	Weight
Biomass	0.37	0.10
Abundance	0.13	0.04
Abundance of empty tests	0.21	0.06
Abundance of drilled empty tests	**0.97**	0.10

N = 9 samples for the first two lines and 8 samples for the second two, since the sample from 05.08.2012 had no drill hole counts for smallest empty tests. Significant correlations highlighted in bold. Bonferroni-corrected significance level for 8 comparisons was 0.05/8 = 0.00625.

### Distribution of *Boreotrophon clathratus* and other predators between the cages

By the end of exposure none of the predator exclosure (full) cages contained any *Boreotrophon clathratus* individuals larger than 7 mm. Most adult whelks in enclosure cages survived the experiment, so that these cages finally contained on average 6.3±0.4 live individuals sized 8 mm and larger; partial and open cages had several times fewer. Upon retrieval most cages also contained smaller *B*. *clathratus* (≤7 mm) that apparently could pass through 2.5 mm mesh and were unevenly distributed among the treatments: whelk enclosures and full cages had significantly more than partial and open cages did (Table 4). Each crab enclosure contained one *B*. *clathratus* individual ≤7 mm (Table 4), while a starfish cage contained two.

**Table 4:** Field caging experiment: SS of type III sum of squares ANOVA on abundance of the whelks *Boreotrophon clathratus* by the end of exposure and mortality of adult barnacles *Balanus crenatus* with and without drill holes.

	df	Number of small *Boreotrophon clathratus* (≤7 mm) per cage	Number of large *Boreotrophon clathratus* (≥8 mm) per cage	Total mortality of barnacles ≥5 mm	Mortality of barnacles ≥5 mm without drill holes	Mortality of barnacles ≥5 mm that were fatally drilled	Number of fatally drilled barnacles ≥5 mm per cage	df	Fraction of empty tests ≥5 mm with drill holes ^a^
Transformation		square root	none	none	none	none	square root		none ^b^
Source of variation									
Treatment (fixed)	3	12.390**	160.5***	0.481***	0.000ns	0.468***	56.4***	3	2.044**
Year (random)	2	14.254**	3.3ns	0.006ns	0.002ns	0.014ns	0.4ns	-	-
T x Y (random)	6	2.138ns	3.4ns	0.021ns	0.004ns	0.013ns	3.4ns	-	-
Error	12	7.206	3.5	0.056	0.007	0.046	6.8	15	1.279
Means by Treatment level and post-hoc tests									
Whelk enclosure		4.7±1.6 a	6.3±0.4 a	37.2±2.9% a	2.5±1.5% a	34.7±3.1% a	16.2±2.6 a		93.4±3.8% a (n = 6)
Partial cage		1.2±1.0 b	0.5±0.4 b	7.6±3.8% b	2.2±0.8% a	5.4±3.1% b	1.3±0.7 b		51.3±18.3% ab (n = 4)
Open cage		1.0±0.8 b	0.7±0.3 b	5.1±1.9% b	2.3±0.9% a	2.8±2.3% b	0.8±0.5 b		33.3±16.7% b (n = 6)
Full cage		5.7±2.8 a	0.0±0.0 b	1.8±1.1% b	1.8±1.0% a	0.0±0.0% b	0.0±0.0 b		0.0±0.0% b (n = 3)
Crab cage^c^		1.0±0.0	0.0±0.0	1.2±1.2%	1.2±1.2%	0.0±0.0%	0.0±0.0		0.0 (n = 1)

Empty barnacle tests with complete through drill holes considered 'fatally drilled'. Variables transformed to achieve homogeneity of variances where needed. The results of Tukey HSD post-hoc tests are indicated by letters 'a' and 'b' following the means. Significantly different means have no letter in common.

**–p < 0.01

***–p < 0.001

ns–not significant.

^a^ One-way ANOVA performed since many cages had no empty tests ≥5mm.

^b^ Variances were heterogeneous regardless of the transformation used; no correlation between means and variances detected.

^c^ Supplementary treatment not included in the analyses due to poor replication (1 cage in 2010–2011 and 2 cages in 2011–2013).

Small barnacles (<5 mm) were highly abundant in full cages throughout the experiments, in open cages in 2011 and 2012, and in whelk enclosures in 2012, showing no consistency with the pattern found in small whelks.

Per square meter, both partial and open cages on average had more *B*. *clathratus* (14.81±9.92 and 14.81±8.79 individuals, respectively) than we observed in the field (see above), but the variation was high and the difference was insignificant (Student T-test). Their biomasses (6.117±2.795 and 3.673±2.615 g·m^-2^, respectively) were more similar to field values and also highly variable. The *B*. *clathratus* to *B*. *crenatus* weight ratios were 0.016±0.009 in partial cages and 0.004±0.003 in open cages, both insignificantly different from the field average (0.005±0.002; Student T-test).

We found adult and juvenile spider crabs *Hyas araneus* in every partial cage, on average 2.5±1.0 individuals with total weight 1.26±0.70 g per cage. Some whelk and exclosure cages contained *H*. *araneus* as well, but only the juveniles (1.0±0.8 ind., 0.01±0.01 g and 0.5±0.3 ind., 0.01±0.01 g per cage, correspondingly). Also, most partial cages contained shrimps *Spirontocaris phippsi* (8.3±3.1 ind., 0.86±0.45 g per cage) and *Eualis gaimardi* (3.3±1.6 ind., 1.63±0.72 g per cage) as well. *S*. *phippsi* (but not *E*. *gaimardi*) juveniles were also more or less frequent in whelk and exclosure cages (1.7±0.7 ind., 0.04±0.02 g and 7.7±3.9 ind., 0.11±0.06 g per cage, correspondingly). Because of the high mobility of crabs and shrimps we did not record their abundance in open cages.

### The effect of *Boreotrophon clathratus* on *Balanus crenatus*

**Fig 3:**
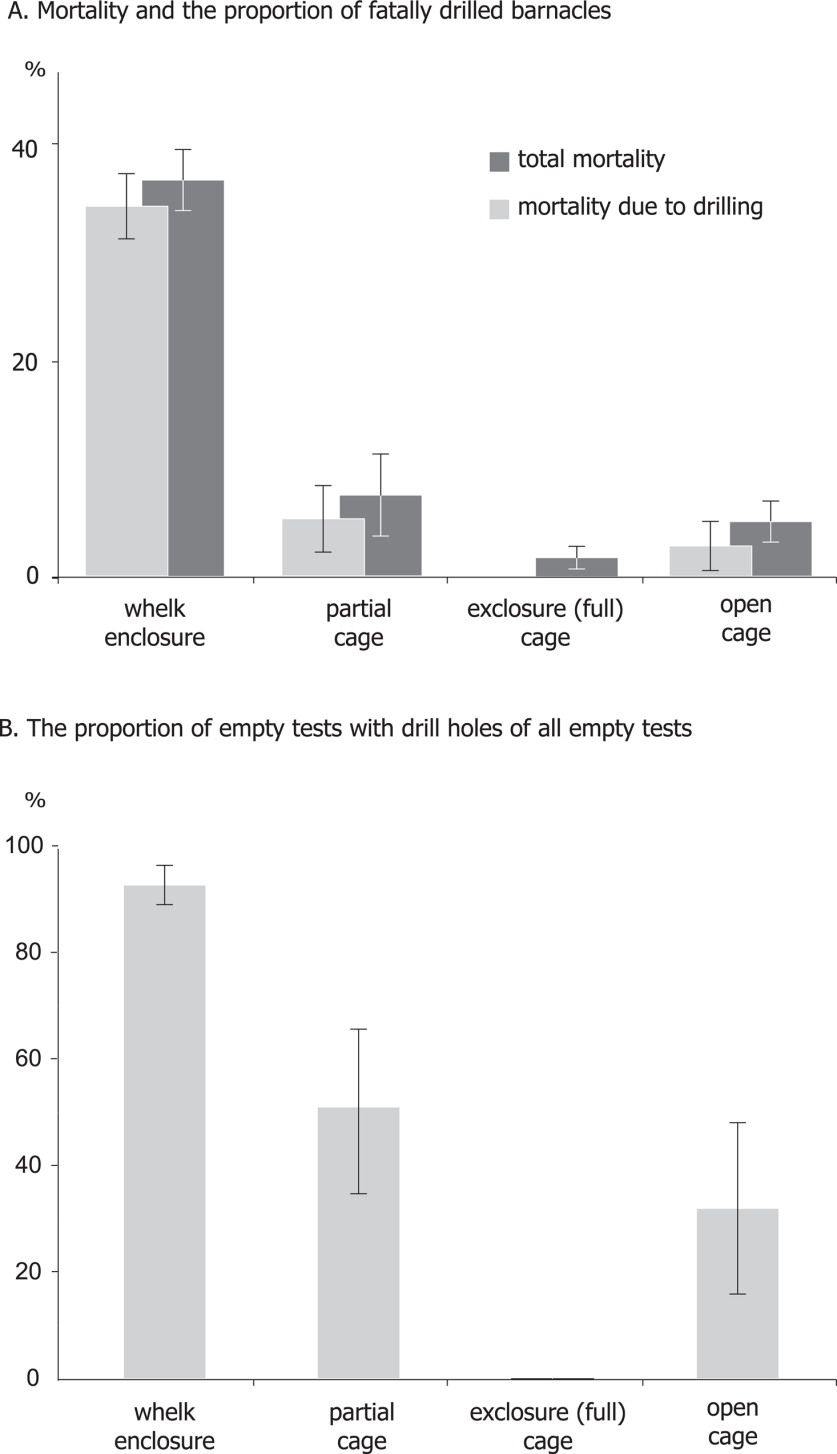
Contributions of drilled and intact empty tests in total mortality of barnacles in different treatments. (A) Total mortality of adult barnacles and the proportion of fatally drilled ones. (B) The proportion of drilled empty barnacle tests of total number of empty tests by the end of the experiment. Barnacles with aperture length not less than 5 mm were taken into account. Error bars denote S.E.

According to photographs, every barnacle ≥5 mm initially placed in a cage by the end of the experiment was either found live or turned into an empty test; no individual was lost untraced. The proportion of empty tests in adult barnacles was thus equal to mortality. Presence of adult *B*. *clathratus* strongly affected the survival of adult barnacles (Table 4). At the end of the experiments 37±3% of adult barnacles in whelk treatments and 8±4%, 2±1% and 5±2% in partial, full and open cages, respectively, were found dead. Empty tests they left had no single drill hole in the full cages. In contrast, 93±4% of the empty tests ≥5 mm in the whelk enclosures were drilled at least once, whereas partial and open cages had 51±18% and 33±17% empty tests ≥5 mm drilled (Fig 3). While the contribution of drilled empty tests to mortality of barnacles ≥5 mm was significantly higher in whelk enclosures than in any other treatments, the contribution of non-drilled empty tests was around 2% in any cages and was not affected by treatments (Table 4).

There were no empty barnacle tests in the only starfish cage by the end of the experiment. Out of the three crab cages one had 2 empty tests ≥5 mm without drill holes, and the other two had no empty tests (Table 4).

**Fig 4:**
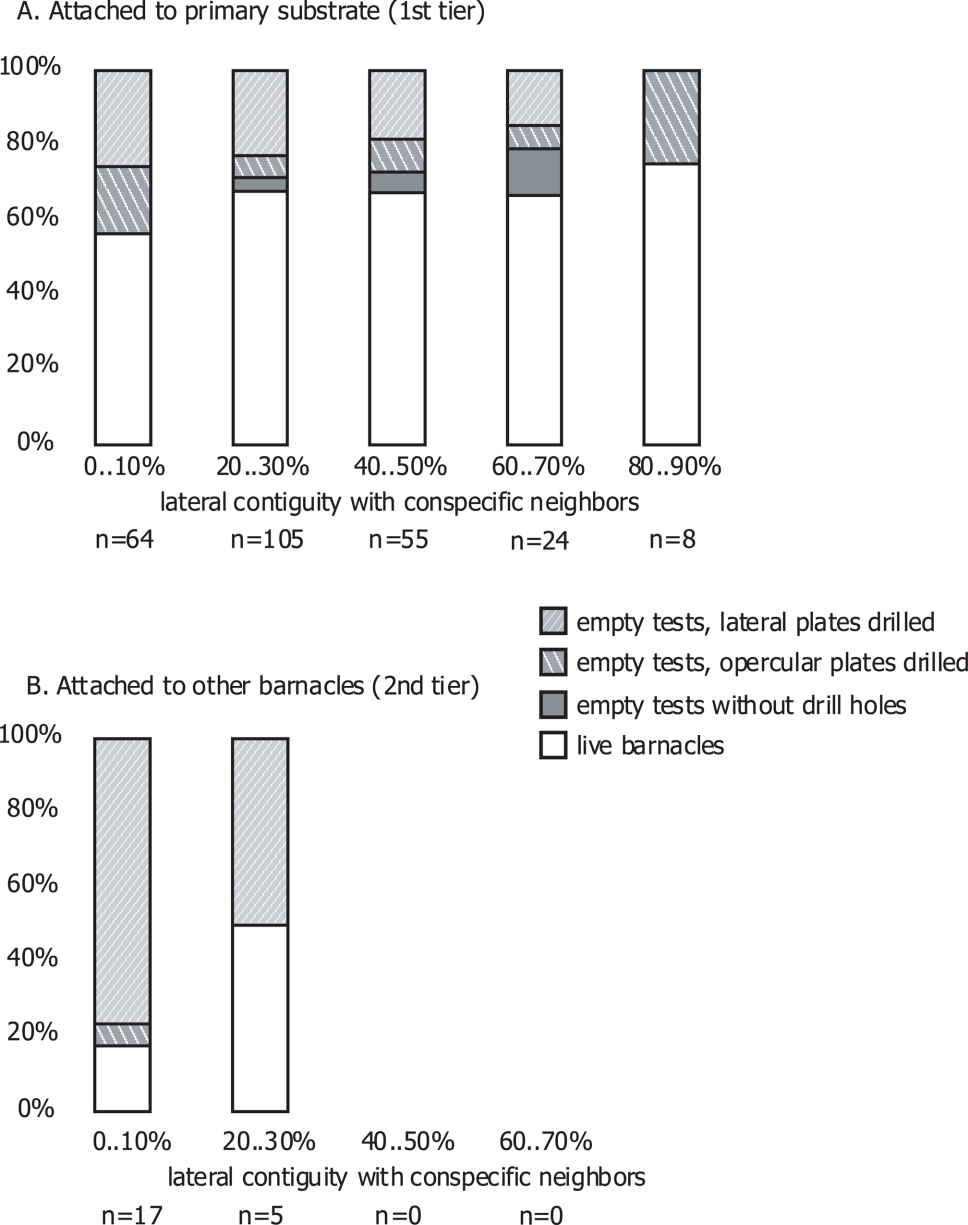
Differential survival of barnacles in predator enclosure cages. Survival of adult barnacles by tier and lateral contiguity with conspecific neighbors. Pooled data for 6 whelk enclosure cages.

Nearly all the empty tests in the whelk enclosures had their opercular plates completely preserved. The survival rate in the whelk enclosures was higher (pairwise Student T-test p = 0.003) in the 1^st^ tier barnacles attached to primary substrate (66±3%) than in those growing on conspecifics (13±8%), which had less lateral contiguity with neighbors (Fig 4). These 2^nd^ tier barnacles also had been primarily (94±6%) attacked through their wall shell plates, while the empty tests from the 1^st^ tier had 33±5% of drill holes on the opercular plates (Fig 4).

**Fig 5:**
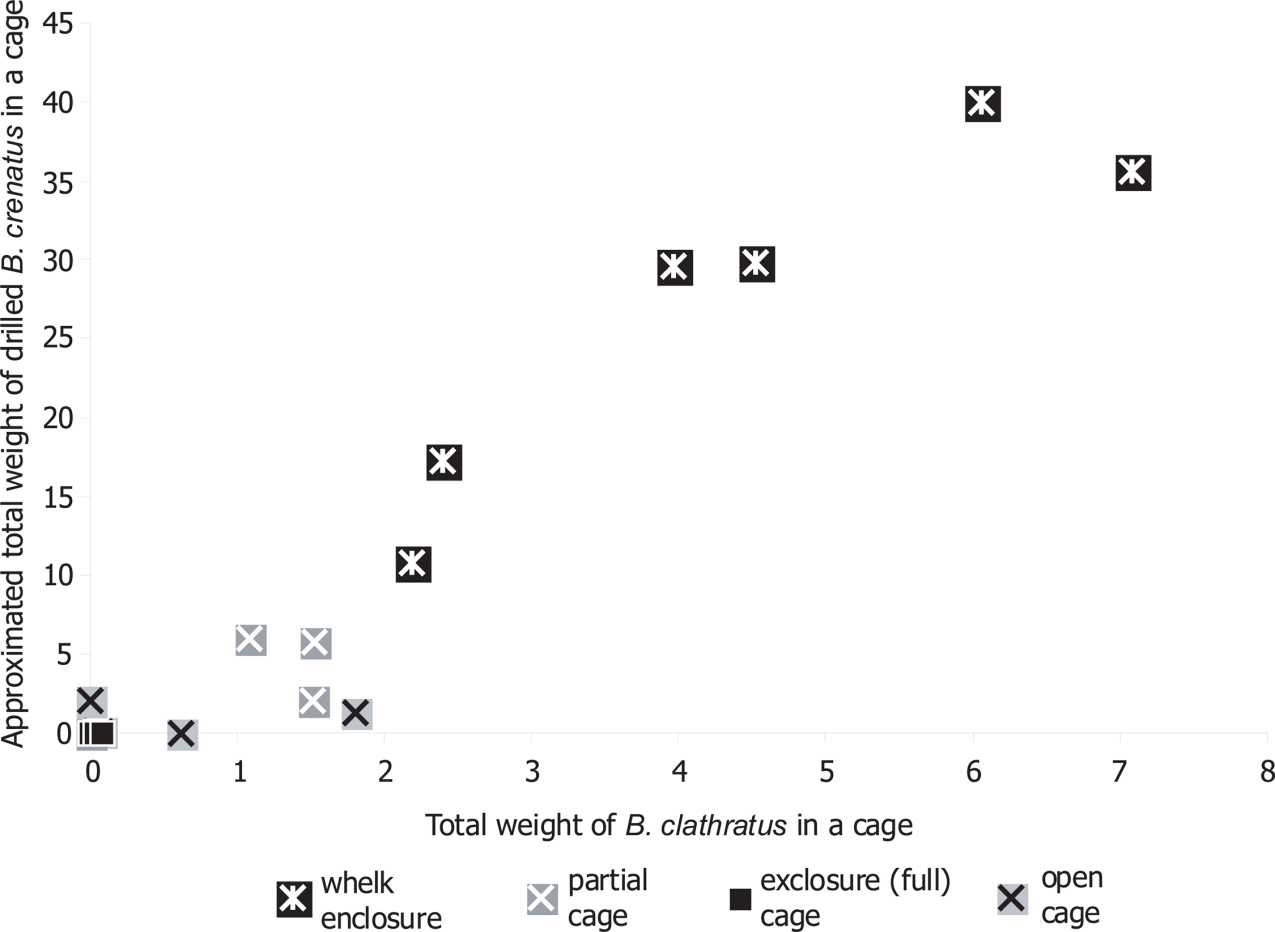
Weight of predators vs weight of prey consumed. The relationship of the weight of *Boreotrophon clathratus* and approximated weight of fatally drilled *Balanus crenatus* by cage in the field experiment.

According to the estimates of barnacle individual weight based on the size of empty tests they had left, total weight of *B*. *clathratus* found in a cage positively correlated (R = 0.96, p = 0.000, n = 24) with the approximated total weight of the fatally drilled *B*. *crenatus* across all the cages and treatments (Fig 5). In particular, within the enclosure cages *B*. *clathratus* consumed 6.3±0.4 times more barnacles than they themselves weighed (correlation of the predator and prey weights was R = 0.92, p = 0.010, n = 6). Average per capita consumption was 2.5±0.3 barnacles ≥5 mm. Average size and weight of the whelks >7 mm varied between enclosure cages. Smaller whelks weighing 0.4 g (17 mm high) and larger ones weighing 0.7–0.9 g (20–22 mm high) consumed about 1.5 and 3 adult barnacles per year, respectively.

### Assemblages associated with live *Balanus crenatus* and their empty tests

The macrobenthic assemblage that developed within the patches of initially defaunated barnacles and their empty tests after a year of exposure was numerically dominated by juveniles of relatively long-living sessile organisms: a bivalve *Heteranomia squamula*, *Balanus crenatus* recruits, red algae seedlings (likely *Phycodrys rubens*, *Odonthalia dentata* and *Ptilota gunneri*), ascidians *Styela* spp., and a mobile bivalve *Musculus discors*. In total, we identified 89 taxa (most to the species level), including 32 polychaets, 19 bryozoans, 9 bivalves, 7 amphipods and 5 gastropods, and found 73 species in LB and 65 –in ET, of which 49 were common in both treatments. The exposure time was too short to determine the exact species of most slow-growing taxa like ascidians and red algae.

**Fig 6:**
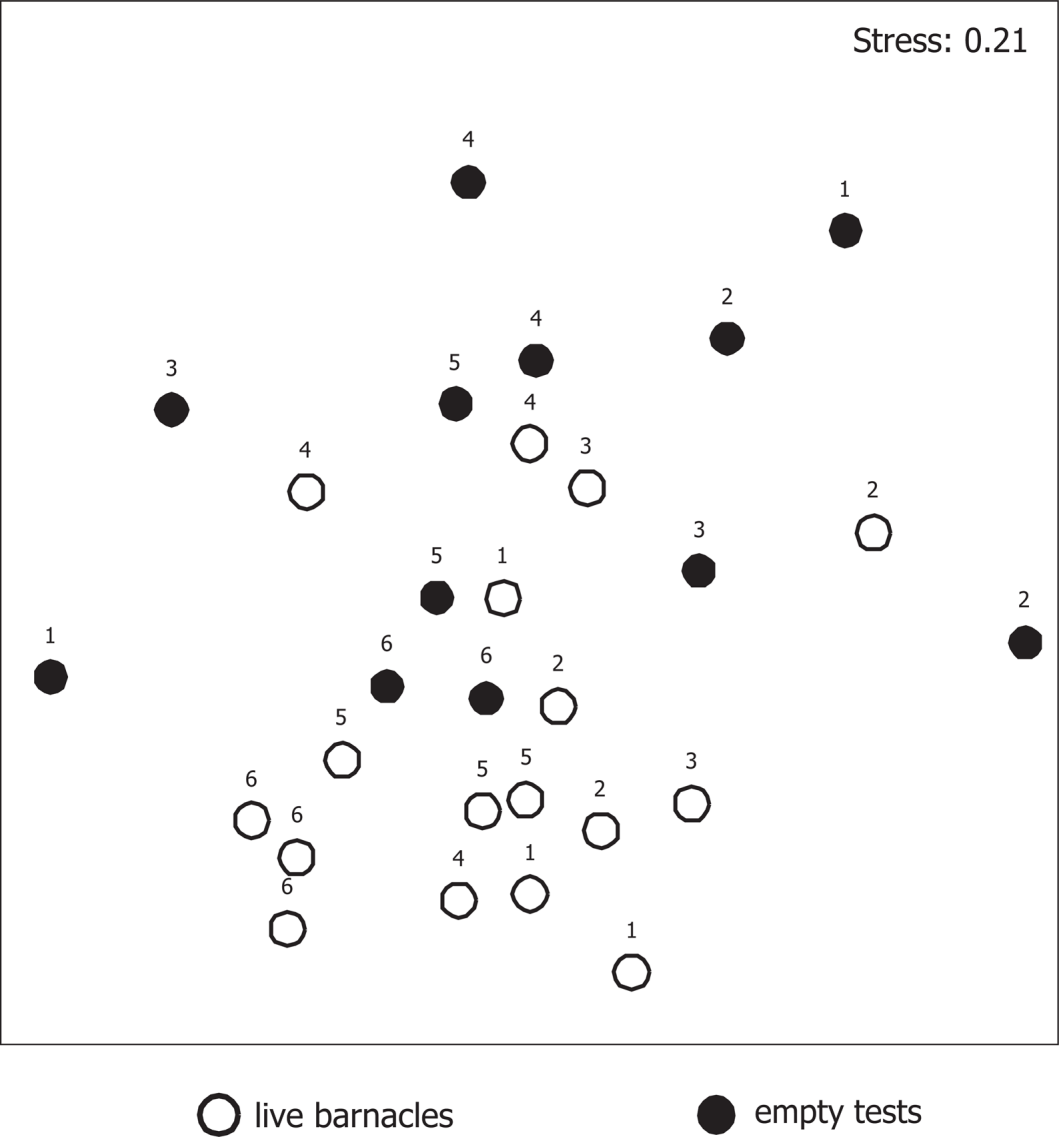
Macrobenthic assemblages associated with live barnacles and their empty tests after 1 year of exposure. Non-metric MDS on fourth root transformed abundances of 15 most important species standartized by the number of adult barnacles or their empty tests on a substrate. Bray-Curtis similarity. Labels indicate the grid number.

Average species richness and diversity were similar in LB (20.5±1.4, 2.2±0.1) and ET (19.2±1.9, 2.0±0.1, respectively) treatments (2-way type III sum of squares ANOVA with factors Live [LB vs ET, fixed] and Grid [random] followed by Tukey HSD post-hoc tests). Pooled diversity for the whole sample was 2.7 (2.6 for pooled LB and 2.7 for pooled ET). The assemblages that developed in LB and ET were markedly different (Table 5, Fig 6). Particularly, LB had more barnacle recruits and juvenile discord mussels *Musculus discors*, as well as mobile polychaets *Pygospio elegans* and *Cirratulis cirratus*. A sessile tubeworm *Circeis armoricana* and a bryozoan *Stomacrustula cruenta* were associated with ET (Table 6).

**Table 5:** Field experiment: multivariate comparison of macrobenthic assemblages associated with live barnacles (LB) and their empty tests (ET) after 1 year of exposure.

Source of variation	df	SS	MS	pseudo-F	p (perm)	Unique permutations
Live (LB and ET, fixed)	1	2521	2521	2.121	**0.036** *	4965
Grid (1. . .6, random)	5	9905	1981	1.905	**0.004** **	4958
Live x Grid (random)	5	5946	1189	1.144	0.218ns	4960
Error	17	17679	1039			

Type III sum of squares PERMANOVA results for 5000 permutations on fourth root transformed abundances standardized by the number of adult barnacles or their empty tests on a substrate. Bray-Curtis similarity. Unrestricted permutation of raw data.

*–p < 0.05

**–p < 0.01

ns–not significant.

**Table 6:** Field experiment: the percentage contribution (individual C% and cumulative S%) of different species to average measures of dissimilarity (D) between the macrobenthic assemblages associated with live barnacles (LB) and their empty tests (ET) after 1 year of exposure, and their average abundance in LB and ET.

			SIMPER results			Average abundance (ind. per adult barnacle, untransformed)		SS of the type III sum of squares 2-way ANOVA with Grid (1.6) and Live (LB and ET) effects				
Species	T	M	D	C%	S%	Empty tests	Live barnacles	Grid (random, df = 5)	Live (fixed, df = 1)	Live x Grid (random, df = 5)	Error (df = 17)	Tr
*Musculus discors*	m	mo	2.29	4.5	4.5	0.04±0.02	**0.46±0.10**	0.55ns	1.55*	0.51ns	1.85	frt
Red algae seedlings	a	se	2.10	4.1	8.5	1.20±0.37	0.69±0.20	6.68ns	1.79ns	6.81ns	14.16	none
*Pygospio elegans*	p	mo	1.69	3.3	11.8	0.02±0.01	**0.13±0.04**	0.43ns	0.78**	0.20ns	1.25	frt
Gammaroidea f. gen. sp.	a	mo	1.66	3.2	15.0	0.33±0.19	0.10±0.06	1.41ns	0.11ns	1.17ns	1.90	frt
*Balanus crenatus* <5mm	c	se	1.62	3.2	18.2	0.62±0.21	**1.17±0.22**	12.20**	**1.96** *	0.90ns	5.49	none
*Corophium bonelli*	c	mo	1.51	2.9	21.1	0.11±0.05	0.12±0.05	0.41*	0.00ns	0.06ns	0.45	none
*Munna* sp.	c	mo	1.49	2.9	24.0	0.11±0.04	0.12±0.04	0.05ns	0.00ns	0.13ns	0.51	none
*Dulichia spinosissima*	c	mo	1.49	2.9	26.9	0.13±0.06	0.11±0.05	0.18ns	0.01ns	0.37ns	0.61	none
*Circeis armoricana*	p	se	1.34	2.6	29.5	**0.08±0.02**	0.01±0.01	0.92ns	0.53*	0.33ns	0.51	frt
*Hiatella arctica*	m	mo	1.30	2.5	32.0	0.10±0.04	0.07±0.02	0.19**	0.01ns	0.01ns	0.08	none
*Stomacrustula cruenta*	b	se	1.29	2.5	34.5	**0.15±0.03**	0.04±0.01	0.31ns	0.42**	0.06ns	1.02	frt
*Dendrobeania fruticosa*	b	se	1.28	2.5	37.0	0.07±0.02	0.08±0.02	0.04ns	0.00ns	0.05ns	0.07	none
*Styela* spp.	t	se	1.22	2.4	39.4	0.47±0.12	0.63±0.11	0.49ns	0.13ns	1.63ns	2.63	none
*Cirratulis cirratus*	p	mo	1.21	2.3	41.7	0.02±0.02	**0.07±0.03**	0.04*	**0.02** **	0.01ns	0.21	none
*Capitella capitata*	p	mo	1.18	2.3	44.0	0.07±0.02	0.07±0.02	0.03ns	0.00ns	0.05ns	0.09	none
*Boltenia echinata*	t	se	1.11	2.2	46.2	0.04±0.01	0.03±0.01	0.03ns	0.00ns	0.01ns	0.03	none
*Escharella* sp.	b	se	1.08	2.1	48.3	0.17±0.06	0.27±0.04	0.41ns	0.06ns	0.16ns	0.39	none
*Schizomavella lineata*	b	se	0.96	1.9	50.2	0.01±0.01	0.04±0.01	0.24ns	0.06ns	0.23ns	1.18	frt

SIMPER on fourth root transformed abundances standardized by the number of adult barnacles or their empty tests on a substrate and Bray-Curtis similarity. ANOVA on raw abundances transformed to acheive homogeneity of variances where needed (Tr). T–taxonomic group. M–mobility. Significant differences between LB and ET and significantly higher mean abundances highlighted in bold. mo–mobile, se–sessile, a–algae, b–bryozoans, c–crustaceans, m–molluscs, p–polychaetes, t–tunicates, frt–fourth root transformation,

*–p < 0.05

**–p < 0.01

ns–not significant.

Both absolute and relative mean dry weight of the accumulated soft sediment per experimental substrate were significantly higher (Student T-test, p = 0.008 and 0.000, respectively) in ET (3.53±0.50 g and 10.7±1.3%, respectively) than in LB (1.96±0.23 g and 3.7±0.4%, respectively).

## Discussion

Below, we first address how adult *B*. *clathratus* affected adult barnacles, and estimate the predation strength as detected by the experiments and field observations. Since we conclude that the predation was primarily limited by the abundance of *B*. *clathratus*, we focus further discussion on the factors which may affect this abundance, based on the distribution of juvenile *B*. *clathratus* between caging treatments. Finally, we analyze the community-wide effect of empty barnacle tests as an indirect consequence of the predation.

### The relationship of *Balanus crenatus* and *Boreotrophon clathratus* and the strength of the predation

The results of the caging experiments were consistent with field observations and provided the clear evidence that *B*. *clathratus* preyed on *B*. *crenatus*, while the drill holes observed in empty tests identify past attacks. Both observations and experiments displayed several times higher vulnerability of the barnacles from the second tier (living on conspecifics) as compared to the ones from the first tier (living on primary substrate). At our research sites empty tests constituted about one fourth of the total barnacle cover. Control treatments did not reveal any source of adult barnacle mortality which would not leave empty tests as a trace. According to the drill holes count, in the field *B*. *clathratus* accounted for 7% of barnacle mortality. Although most opercular plates from the empty tests found in the field were lost, this could not have substantially affected our estimates of predation strength. Indeed, according to the caging experiment results, these were the barnacles from the first tier, that suffered most attacks through opercular plates. First-tier barnacles in the field were, however, much less affected by *B*. *clathratus*, compared to the second-tier barnacles, which in the experiments were typically attacked through better preserving lateral walls. As a result, the estimate of adult barnacle mortality whelks account for, corrected by the tier-specific frequencies of opercular attacks obtained from the experiment, is 7.6%.

The contribution of predation by *B*. *clathratus* to the total mortality of *B*. *crenatus* was much higher in experimental partial and open cages (up to 51%) than its estimates from the field observations. Only a small part of this difference could be explained by the loss of opercular plates in the field. Yet, drilled empty tests, being mechanically weakened, may exhibit lower long-term preservation rates than intact ones, resulting in increased accumulation of the latter. For bivalve shells it has been experimentally proved that valves with drill holes degrade faster [29]. Our experimental cages and field samples had the similar biomass of barnacles per square meter, so that the difference in abundance could not affect their availability for predators. However, live barnacles in the field samples (but not in the experiments) were partially covered by epibionts and jumbled with empty tests, and both these factors could reduce predation strength. Attached sessile organisms can protect their hosts from predators by making prey less attractive or accessible for consumers [30]. At the same time, epibionts can directly increase the mortality of their hosts due to overgrowth and consequent feeding interference [31]. Moreover, in the White Sea the abundance of co-dominating solitary ascidians, that often occur on barnacles, is positively correlated with the number of empty tests [10]. Ascidians are likely either to increase barnacle mortality [11] or stabilize their empty tests and protect them from destruction (as red algae do, see [32]), or both. This might also reduce the proportion of drilled tests in the field compared to caging experiments. Importantly, all the abovementioned factors only lead to underestimation of the field predation strength from drill hole counts, for which about 7% of the total mortality is thus a minimal estimate.

Given the predation rate *B*. *clathratus* exhibited in the enclosure cages (6.3 of its own weights per year), in the field they are on average capable of consuming only about 3% of the existing barnacle biomass per year (with a highest estimate of 10% for a sample from 03.08.2013, see Table 1). There is thus no sign that the observed abundance of the predator is limited by prey availability. Foraging of muricids on barnacles had been extensively studied only in tropical and temperate rocky intertidal. In Northern California, depending on the size and species of the prey, *Nucella ostrina* accounts for 34–83% of barnacle mortality [16]. The predation strength of *N*. *lapillis* in New England intertidal varies from 0 to 100%, affected mostly by wave exposure, intertidal level, and algal canopy. The strong control of predation rate by environmental variables in intertidal leads to the absence of correlation between predation intensity and predator abundance [33]. In contrast, our data show that lower predation strength in low-disturbance subtidal is primarily limited by predator abundance, which strongly correlates with the number of drill holes observed in the field (Table 3).

Similarly to other barnacles [34], *B*. *crenatus* in the White Sea experiences heavy recruitment events far exceeding the habitat capacity in terms of hard substrate space [35]. While in intertidal habitats the elevation of sessile organisms above the substrate is strongly limited by wave exposure, subtidal barnacles can avoid space limitation by using the surface of conspecifics and developing multi-tier clusters. As indicated both by our field observations and experiments, the absence of close neighbors (which otherwise screen lateral test walls from intrusion) in second-tier barnacles increases the probability of predation by *B*. *clathratus*. This, in turn, should raise the relative competitive ability of other sessile organisms that use barnacles as a substrate, and contribute to maintaining the observed spatial structure of the epibenthic patches with barnacles monopolizing the primary substrate and hosting co-dominating ascidians and red algae in top tiers [10]

Although it appears that at our research sites *B*. *clathratus* (and also perhaps a much less abundant *B*. *truncatus*) are the only barnacle consumers that leave drill holes, there could be other predators whose attacks do not have easily identifiable traces on empty tests. For instance, it takes several hours for the nudibranch *Onchidoris bilamellata* to break into an adult barnacle by pushing opercular plates inside [36]. However, we only had observed a couple of small *Onchidoris* sp. individuals at the Site 1 back in 2002 and never found any since, despite the numerous samples taken. Yet, given that we only obtain samples in July-August and *O*. *bilamellata* may have a sub-annual lifespan and is capable of mass subtidal migrations [37], we might underestimate its abundance and impact on barnacles.

Much less likely, barnacles may suffer predation from the buccinid whelks *Buccinum* spp. and *Neptunea despecta* which locally are several times less abundant than *Boreotrophon*. To our knowledge, there only exists a single indirect confirmation of a buccinid preying on barnacles ([38], based on stomach content analyses), and the known diets of *Buccinum* and *Neptunea* species do not include barnacles [39–40]. Finally, some species of starfishes can prey on barnacles [41], but the only species found in our samples, *Henricia* sp., is considered to be either microphagous or sponge-grazing one [42]. In addition, these starfishes did not cause any loss of *B*. *crenatus* ≥5 mm in the only enclosure cage we exposed them in. We thus do not believe that buccinids or starfishes contribute to mortality of adult barnacles at our researh sites.

Although *B*. *crenatus* is likely to be the principal food source for adult *B*. *clathratus*, the diet of the latter is hardly limited to the former. Many empty bivalve shells found in soft sediment samples obtained nearby also have drill holes, which had been initially attributed to naticid predation. Naticid snails, though, are extremely scarce here, while the fraction of drilled shells is up to 69% depending on a prey species [43]. Given our present results, *B*. *clathratus* should be considered a probable consumer of these bivalves. We have also occasionally observed similar drill holes on empty gastropod shells and spirorbid worm tubes. Feeding habits and preferences of *B*. *clathratus*, which may also shift with age, are worth further experimental investigation. Yet, our data clearly indicate that the effect of these whelks on adult barnacles within the community studied is limited by their abundance and not by that of the prey.

### Abundance of *Boreotrophon clathratus*

According to their distribution among the treatments by the end of the experiment, *B*. *clathratus* sized 7 mm and smaller could pass through the 2.5 mm mesh. These juvenile whelks were 5–6 times more abundant in adult whelk enclosures and exclosures than in partial and open cages (Table 4). Technically, this low abundance in open cages could in part result from a sampling artifact, since these were the only treatments without mesh, which might have prevented juvenile whelks from dropping out when we collected them in the other cages. Yet, their similar or lower abundance in partial cages and field samples renders this bias, in case one exists, as unimportant. Trapping of juvenile *B*. *clathratus* by mesh (i.e. easily pass through the mesh when small, and not able to get out as grow further) also does not fully explain the results, since one would lead to intermediate and high abundances in partial and crab cages, respectively. Consequently, the outcome of our experiment provides the evidence that the abundance of juvenile *B*. *clathratus* is controlled by a top predator or predators, either via direct consumption or indirect non-consumptive effects.

*Boreotrophon* spp. has been occasionally reported as a diet component for the common eider [44–45] and wolffish [46]. According to our results, the low abundance of the small whelks in supplementary spider crab treatments suggests *Hyas araneus* could also be one of their consumers. Feeding habits of the spider or toad crabs *Hyas* spp. are unclear despite the attempts of experimental investigation [7], but they are likely to be omnivores and can definitely consume molluscs [47]. The reduction of juvenile abundance that we observed in the experiment predicts that in absence of the top-down control by predators *B*. *clathratus* would account at least for 35–40% of *B*. *crenatus* mortality.

Keystone predation on a foundation species is commonly top-down controlled, reducing the impact on a prey. For instance, sea otters of Aleutian Islands forage on sea urchins and thus reduce their grazing pressure on kelp [48]. Similarly, the predation on herbivorous crabs on salt marshes of New England limits its otherwise fatal grazing on the habitat-forming cordgrass [49]. Temperate rocky intertidal habitats can support multi-level trophic cascades with gulls preying on crabs, which consume dogwhelks, which in turn feed on mussels [50]. In our case, spider crabs that could likely prey on juvenile *B*. *clathratus* must also support more trophic levels above them, as they are reportedly consumed by the bearded seal *Erignathus barbatus* [51], the shorthorn sculpin *Myoxocephalus scorpius* [46], the cod *Gadus morhua* [52], and the common eider *Somateria mollissima* [53].

### Assemblages associated with live barnacles and their empty tests

Regardless of any predation pressure thereon, we found *B*. *crenatus* together with their empty tests entirely dominating the primary hard substrates on the bottom. In the experiment, empty tests accumulated more soft sediment particles than live barnacles. While species richness and diversity in the patches of initially defaunated live barnacles and empty tests were the same, species composition and relative abundances were different between these microhabitats. Despite the higher capacity for the accumulated sediment particles, empty tests were not enriched with purely infaunal species. Importantly, barnacle recruits were twice more abundant on live adult conspecifics than on their empty tests. This result is consistent with previous field observations on the local distribution of *B*. *crenatus* by substrate type [10]. In the field, however, empty tests are often associated with co-dominating large perennial solitary ascidians, so that their effects on the associated fauna are hardly distinguishable without manipulations [10,11,14].

Macrobenthic assemblages associated with empty barnacle tests had been mostly studied in intertidal. In rocky intertidal habitats of New South Wales (Australia) and northern California empty tests similarly shelter mobile organisms from desiccation and wave exposure [16,32]. On Australian rocky shores numerous species of snails, polychaetes, mites, amphipods and flatworms are more abundant inside empty tests than among live barnacles [12]. In contrast, in shallow subtidal of Tampa Bay (Florida) the experimental removal of soft tissues from barnacles has little effect on the total abundance, species richness and composition of the associated fauna [54]. It would be generally expected, if in the absence of harsh environmental stress live and dead barnacles had similar effects on the associated organisms. For instance, in the White Sea subtidal, adding simple structures made of PVC tubes to the bare soft sediment assembles the species composition rather similar to the one observed within the patches of live barnacles [55]. Yet, our present data show that the effect of barnacles in a subtidal habitat is far from purely architectural, and the biogenic activity of the live ones does make the difference.

In temperate rocky intertidal habitats, where barnacles develop a single tier on a primary substrate surface, empty tests can strongly enhance their recruitment compared to bare rock [13,17]. The barnacle mortality sources which leave their empty tests holding the substrate space (like predation by whelks) may thus cause an indirect positive effect on barnacle population unlike those destroying the tests (e.g. dislodging by wave action). In the White Sea subtidal more than 90% of the smallest *B*. *crenatus* are found either on primary substrate or live conspecifics, despite the constant presence of numerous empty tests [10]. Our present study provides the evidence that the patches of empty tests are much less favourable for their recruits compared to those of live barnacles. Consequently, the negative effect of predation on barnacle population here is additionally amplified by the following decrease in recruitment until the empty tests collapse and release primary substrate space, which may take years.

## Conclusions

We found that in the White Sea subtidal *B*. *clathratus* feeds on *B*. *crenatus* by drilling holes in their shell plates. Empty barnacle tests supported a markedly different assemblage of macrobenthic organisms compared to live barnacles and attracted twice less barnacle recruits. At the sites studied *B*. *clathratus*, however, was scarce and made a minor contribution to the empty tests supply, accounting for about 7% of barnacle mortality. Unlike in tropical and temperate intertidal, the predation strength was limited by predator density, not by disturbance level and prey availability. Given the relatively high abundance of small *B*. *clathratus* individuals in predator exclosure cages, it is likely that this species is in turn controlled by top level predators, which limits its effect on barnacle population. Though apparently less strong than in tropical and temperate waters, predation in arctic subtidal causes the indirect effects not quite predictable based on the results from previously studied systems. Further experiments are needed to reveal the processes that control the community structure in high-latitude complex subtidal habitats, so far largely unexplored.

## Supporting Information

S1 File Data Field Sampling. This file contains the detailed data on individual barnacles *Balanus crenatus*, their empty tests and whelks *Boreotrophon clathratus* sampled in the field for this study, as well as on dates and field localities of the samples.
(XLS)

S2 File Data Caging Experiment. This file contains the detailed data from the caging experiments with barnacles *Balanus crenatus* and whelks *Boreotrophon clathratus* used in this study.
(XLS)

S3 File Data Empty Tests Experiment. This file contains the detailed data from the experiment on colonization of initially defaunated live barnacles *Balanus crenatus* and their empty tests used in this study.
(XLS)
